# Robust information transfer calculation reveals dynamic networks during sleep arousal

**DOI:** 10.3389/fnetp.2026.1930617

**Published:** 2026-09-17

**Authors:** Richard Hohmuth, Jakob J. Müller, Hagen Malberg, Martin Schmidt

**Affiliations:** Institute of Biomedical Engineering, TU Dresden, Dresden, Germany

**Keywords:** autonomic regulation, cardiovascular disease, heart rate variability, information dynamics, network physiology, sleep arousals, sleep heart health study, transfer entropy

## Abstract

Understanding the influence of cardiovascular diseases (CVD) on human regulatory functions is a major challenge in modern healthcare. This study explores whether the autonomic response to sleep arousals can serve as a diagnostic marker of the health of physiological networks. We introduce a novel method for the robust calculation of information transfer, based on the optimization of embedding parameters for transfer entropy (TE) calculation. Using this approach, we analyzed the information transfer dynamics of 8,136 sleep arousals from 1,956 subjects from the SHHS follow-up visit. Compared to three state-of-the-art approaches, our newly introduced method, max. TE, achieved a 17.9% more stable TE estimate than the second-best approach. Our results indicate a significantly (p<0.001) lower TE in patients with CVD ( 85.4±0.8 mnats) than that in healthy controls ( 87.9±0.5 mnats), indicating an impairment of regulatory control mechanisms. Furthermore, the TE decreases linearly with increasing age and shows significant differences between sleep stages, with the highest TE occurring during REM sleep (p<0.001) . These findings demonstrate that sleep arousals, as a physiological stimulus, can reveal sleep stage- and disease-related changes in cardiovascular networks and that our approach provides a robust group-level embedding strategy for physiological information-transfer analysis.

## 1 Introduction

The human body is a complex network of physiological systems that requires information transfer to control regulatory mechanisms (Ivanov, 2021). Furthermore, human physiology can deviate under different human states, with each state enabling the physiological response required to return to a state of homeostasis. Homeostasis is defined as the state in which physiological processes are in balance. In terms of information transfer, this state requires the least amount of information transfer because homeostasis is an energy-efficient state (Betts et al., 2022). Coupling between two physiological systems (e.g., organ systems) is indicated by information transfer between them. This coupling was conventionally analyzed by investigating information transfer through biochemical pathways (Ivanov, 2021). Information transfer between organ systems forms dynamic networks that change depending on the human state (Ivanov, 2021). In this manuscript, we distinguish between coupling and information transfer. We use coupling as a broad term for statistical or dynamical interdependence between physiological processes. In contrast, information transfer refers specifically to directed predictive information as quantified by transfer entropy (TE): information from the past of a source process that improves prediction of the destination process beyond the information already contained in the destination’s own past (Schreiber, 2000; Wibral et al., 2013; Lizier, 2013). Therefore, TE is interpreted here as directed predictive information transfer, not as proof of direct mechanistic causation (Lizier, 2013; Wibral et al., 2013).

In the investigation of information transfer between organ systems, it is essential to define the state of an organ system. Vital parameter analysis has demonstrated that time series derived from biological signals can be used to represent the state of the respective organ system (Waxenbaum et al., 2025; Berger et al., 1997). During sleep, the cardiovascular system is dynamically regulated by the autonomic nervous system (ANS). This regulation involves a shift between parasympathetic dominance during non-rapid eye movement (NREM) sleep, which promotes cardiovascular rest and a restorative drop in blood pressure, and sympathetic activation during rapid eye movement (REM) sleep, which causes surges in heart rate and blood pressure similar to waking states (Schmidt et al., 2019; Tobaldini et al., 2013; Gurubhagavatula, 2015). A key modulator of this process is sleep arousal, a brief awakening event that triggers a cardiovascular response. Sleep arousals are a spontaneous activation of the central nervous system (CNS), resulting in increased information transfer. Sleep arousals cause an abrupt sympathetic surge and parasympathetic withdrawal, leading to transient spikes in heart rate and blood pressure. While sleep arousals are physiologically protective, such as in terminating apnea events, frequent arousals impose a significant hemodynamic burden and are a primary driver of cardiovascular strain (O’Driscoll et al., 2010; Horner, 1996).

Relationships between physiological feature time series may contain linear and nonlinear dependencies, and these dependencies can change with physiological state (Bashan et al., 2012; Kantz and Schreiber, 2003). Several complementary frameworks have been developed to quantify directional relationships between time series, including Wiener–Granger causality, information-theoretic approaches such as TE and directed information, and nonlinear dynamical-systems approaches based on state-space reconstruction (Granger, 1969; Schreiber, 2000; Massey, 1990; Kantz and Schreiber, 2003; Garfinkel et al., 2017). Classical Granger causality is commonly formulated in terms of predictive improvement, whereas TE provides an information-theoretic measure of directed predictive dependence that can capture nonlinear statistical relationships and is equivalent to Granger causality for Gaussian variables under specific assumptions (Granger, 1969; Schreiber, 2000; Barnett et al., 2009; Wibral et al., 2013). Directed information provides a related information-theoretic framework for directional information flow in systems with feedback (Massey, 1990). Information-theoretic approaches have quantified directional coupling in the CNS, demonstrating information flow patterns in EEG spatiotemporal dynamics through TE measures (Faes et al., 2016). Furthermore, information transfer within the ANS, as reflected by cardiorespiratory interactions, exhibits three independent coupling forms—phase synchronization, amplitude modulation, and time delay stability—revealing dynamic transitions across human states (Bartsch et al., 2014). TE has also been applied directly to cardiorespiratory interaction analysis, including lag-specific cardiovascular and cardiorespiratory information transfer (Faes et al., 2014), heart-rate and breath-rate information transfer in sleep apnea (Schreiber, 2000), and respiration-to-heart-rate information transfer as a potential marker of cardio-respiratory function (Keshmiri et al., 2024). An extended information transfer analysis revealed multi-organ system interactions, indicating that physiological coupling strength varies with human state while maintaining stable brain–brain interactions (Bashan et al., 2012). Recent investigations of autonomic regulation demonstrate that acute mental stress significantly enhances coupling between sympathetic and parasympathetic parameters, indicating adaptive network reorganization under acute mental stress (Hohmuth et al., 2023).

In the presence of cardiovascular diseases (CVD), the regulatory system is fundamentally compromised. CVD is characterized by chronic sympathetic overdrive and reduced parasympathetic tone, which disrupts the bidirectional information transfer between the CNS and the ANS. Afferent signals from the heart to the brain are corrupted by disease-related remodeling, and efferent commands from the CNS are sent through a biased, hypersensitive sympathetic network (Hadaya and Ardell, 2020). It has previously been demonstrated that sleep arousals are a suitable stimulus for the assessment of information transfer, quantified by TE, during sleep (Hohmuth et al., 2024). Utilizing an information-theoretic framework for the investigation of human states is an emerging research field that has revealed changes in information transfer between different human cohorts, such as those differing in age, sex, and CVD status (Bartsch et al., 2014).

Estimating information transfer between coupled systems is a challenging task. State-of-the-art methods depend on the distribution of the vital parameter time series and the underlying coupling mechanisms. Previous research has shown that TE estimation provides promising results for assessing the coupling of biological systems, but it is sensitive to noisy data, and the results are highly dependent on the chosen embedding parameters (Faes et al., 2014; Faes et al., 2016; Hohmuth et al., 2023; Hohmuth et al., 2024). Therefore, various embedding strategies have been developed to identify the optimal embedding for TE estimation between biological systems. The use of information criteria, such as the *Akaike information criterion* (Akaike, 1998) or *Bayesian information criterion* (Stoica and Selen, 2004; Schwarz, 1978), demonstrates the possibility of objectively setting embedding parameters for TE estimation (Faes et al., 2014). Furthermore, the estimated information storage was used to determine optimal embedding parameters for TE estimation. Values such as *Active Information Storage (AIS)* or strategies such as the *Ragwitz criterion* promise good performance in the optimal choice of embedding parameters (Ragwitz and Kantz, 2002). However, these strategies have limitations when applied to TE calculation between physiological systems, for example, between the ANS and CNS. The Ragwitz criterion aims to optimize the prediction of a single time series, while AIS-based strategies optimize the information stored in the past of a single process. Thus, both approaches optimize objectives that are related to, but not identical to, directed source–destination information transfer (Ragwitz and Kantz, 2002; Lizier, 2013). Furthermore, in real-world physiological data, short and noisy windows, finite sample size, and estimator bias can lead to unstable event-wise embedding parameters (Bossomaier et al., 2016; Müller et al., 2016). These limitations are explained in detail in Section 2.2. Therefore, a stable group-level embedding strategy is required to assess information transfer within a large physiological dataset.

In this study, we propose a new optimization technique called *maximum TE optimization (max. TE)* as an information-theoretic framework for assessing coupling in settings with unknown underlying biochemical pathways. In order to validate the proposed concept, the methodology is applied to well-known couplings of the ANS, and the results are compared with those reported in the literature. The methods chapter describes the method optimization and physiological investigation approach. Furthermore, we explain the concept of *max. TE* optimization in detail. In the results chapter, we demonstrate how *max. TE* performs compared to state-of-the-art embedding strategies and how physiological factors, sleep stage, CVD, and demographic factors influence information transfer between the ANS and CNS. In the discussion chapter, we conclude our findings on the optimized TE calculation and describe how sleep arousals reveal the impact of CVD on information transfer between ANS- and CNS-related parameters.

## 2 Materials and methods

### 2.1 Data material and time series extraction

The data originate from the Sleep Heart Health Study follow-up visit (SHHS2) (Zhang et al., 2018; Quan et al., 1997). Electrocardiography (ECG) and electroencephalography (EEG) from 2,154 polysomnograms were examined. The SHHS2 dataset contains summary information on CVD diagnosis. Subjects without a CVD diagnosis were considered healthy controls for this evaluation, even if they had another non-CVD diagnosis. Additionally, the SHHS2 dataset contains annotated sleep events, such as sleep arousals. For further investigation, sleep arousals were used as trigger events to investigate changes in information transfer due to alterations in human state. Therefore, we extracted arousals from the SHHS2 study. Arousals are labeled according to the sleep stage in which they occurred. No distinction was made between transient arousals, where the subject remains in the same sleep stage after arousal, and non-transient arousals, where the sleep stage changes after arousal. Arousals were eligible for our analysis if no other arousal occurred within a 400 s window before or after the arousal. The window size was chosen according to the recommended 300 samples for TE calculation in interaction analysis (Müller et al., 2016), with an additional 100 samples sliding window for parameter extraction at a sampling frequency of 1 Hz. Thus, we ensured that no information about the previous or subsequent arousal was included in the further analysis. In addition, the subject had to be in a stable sleep state according to the SHHS definition during the whole window. A total of 8,136 sleep arousals from the SHHS2 met these criteria and were, therefore, selected for further analysis. Approximately 15.9% of the selected arousals were from subjects with CVD, while 19.5% of the subjects were diagnosed with CVD in the SHHS2. Selected arousals overall came from subjects with similar age ( 60.8±10.4 years vs. 61.9±10.3 years), with a lower apnea-hypopnea-index (AHI) ( 20.3±12.9 events per hour vs. 23.2±15.0 events per hour) and a lower arousal index ( 14.1±6.1 sleep arousals per hour vs. 17.0±8.5 sleep arousals per hour) (see Table 1). The selected arousals comprised 38.5% male and 61.5% female sleep arousals, while the SHHS2 dataset comprised 45.7% male and 54.3% female individuals.

**TABLE 1:** Differences between original patient characteristics (SHHS2) and patient characteristics of selected arousals and the development dataset.

Dataset	SHHS2	Selected arousals	Development	Investigation
No. of arousals	277,821	8,136	400	7,736
No. of subjects	2,154	1,956	336	1,945
Male	981	860	126	853
Female	1,173	1,096	210	1092
Age in years	61.7 ± 10.3	61.4 ± 10.2	60.7 ± 10.4	61.4 ± 10.2
Subjects with CVD	420	356	58	351
Arousal index (events per hour)	17.0 ± 8.5	16.3 ± 7.0	14.3 ± 6.5	16.2 ± 7.0
AHI (events per hour)	23.2 ± 15.0	22.1 ± 14.2	20.4 ± 14.0	22.1 ± 14.2

CVD, cardiovascular diseases; AHI, apnea-hypopnea-index.

The ECG lead corresponds to Eindhoven lead II. Two EEG leads were recorded, where C4-A1 was used for this investigation. EEG and ECG signals were notch-filtered at 60 Hz. Additionally, artifacts from electrical cardiac fields were removed from EEG using the FieldTrip toolbox for MATLAB (Oostenveld et al., 2011). Subsequently, we derived the power of the α , β , γ , δ and θ bands and the Shannon entropy $H_{S}$ from EEG (see Table 2). EEG parameters were derived using the wavelet-based feature extraction approach described by Hartmann and Baumert (2019). To extract beat-to-beat changes in RR and QT intervals, we used the two-dimensional signal warping algorithm (Schmidt et al., 2014; Schmidt et al., 2018). This approach performs piecewise two-dimensional adaptation of a beat template to each individual heartbeat, allowing robust delineation of QRS onset and T end even in the presence of typical ECG artifacts, and has been shown to yield accurate beat-to-beat QT interval measurements (Schmidt et al., 2014). Subsequently, we calculated ten heart rate variability (HRV) and five QT interval variability (QTV) parameters from ECG using sliding windows of 100 s with 99% overlap (see the selected parameters in Table 2). During parameter extraction, records with more than 50% abnormal QT or RR intervals were excluded.

**TABLE 2:** Parameters selected for investigation and their assignment to sympathetic (SNS), parasympathetic (PNS), and central nervous system (CNS).

Parameter class	Parameter	Description	Assignment
Heart rate variability	HR	Mean heart rate	SNS + PNS (Waxenbaum et al., 2025)
	SDNN	Standard deviation of normal-to-normal (NN) intervals	SNS + PNS (Shaffer and Ginsberg, 2017)
	pNN50	Probability of successive NN intervals > 50 ms	PNS (Electrophysiology, 1996)
	RMSSD	Root mean square of successive differences	PNS (Electrophysiology, 1996)
	HF	Absolute power of the high frequency band (0.15 … 0.4 Hz)	PNS (Sammito and Böckelmann, 2021)
	LF	Absolute power of the low frequency band (0.04 … 0.15 Hz)	SNS (Sammito and Böckelmann, 2021)
	VLF	Absolute power of the very low frequency band (0.0033 … 0.04 Hz)	SNS + PNS (Electrophysiology, 1996)
	LF-HF ratio	Ratio between LF and HF frequency bands	SNS + PNS (Sammito and Böckelmann, 2021)
	pLF	Percentage of LF power to total power	SNS (Shaffer and Ginsberg, 2017)
	pHF	Percentage of HF power to total power	PNS (Shaffer and Ginsberg, 2017)
QT variability	QTVN	Normalized QT variance	SNS (Berger et al., 1997)
	QTVi	QT variability index	SNS (Berger et al., 1997)
	QTmean	Mean QT interval length	SNS (Berger et al., 1997)
	STVQT	Short-term variability of the QT interval	SNS (Berger et al., 1997)
	SDQT	Standard deviation of QT intervals	SNS (Berger et al., 1997)
Cortical activity	α -power	EEG band power in 8 … 15 Hz frequency band	CNS (Schomer and Lopes Da Silva, 2017)
	β -power	EEG band power in 15 … 30 Hz frequency band	CNS (Schomer and Lopes Da Silva, 2017)
	γ -power	EEG band power in 30 … 60 Hz frequency band	CNS (Schomer and Lopes Da Silva, 2017)
	δ -power	EEG band power in 0 … 4 Hz frequency band	CNS (Schomer and Lopes Da Silva, 2017)
	θ -power	EEG band power in 4 … 8 Hz frequency band	CNS (Schomer and Lopes Da Silva, 2017)
	$H_{s}$	Shannon entropy	CNS (Schomer and Lopes Da Silva, 2017)

HRV and QTV parameters were selected according to their relationship to the sympathetic nervous system (SNS) and parasympathetic nervous system (PNS), both of which are components of the ANS, as shown in Table 2. Sleep stages and sleep event annotations, including sleep arousals, were provided by the SHHS study. Sleep staging in SHHS was performed by trained polysomnographic technicians following the standardized SHHS scoring protocol based on Kales et al. (1968). Signal quality assessment procedures and inter-scorer reliability of the SHHS scoring pipeline are described in the SHHS study publications (Zhang et al., 2018; Quan et al., 1997) and were used in this work. For further analysis, we combined NREM 1 and NREM 2 into light sleep and NREM 3 and NREM 4 into deep sleep. The sleep stage distribution of the selected arousals is shown in Figure 1.

**FIGURE 1:**
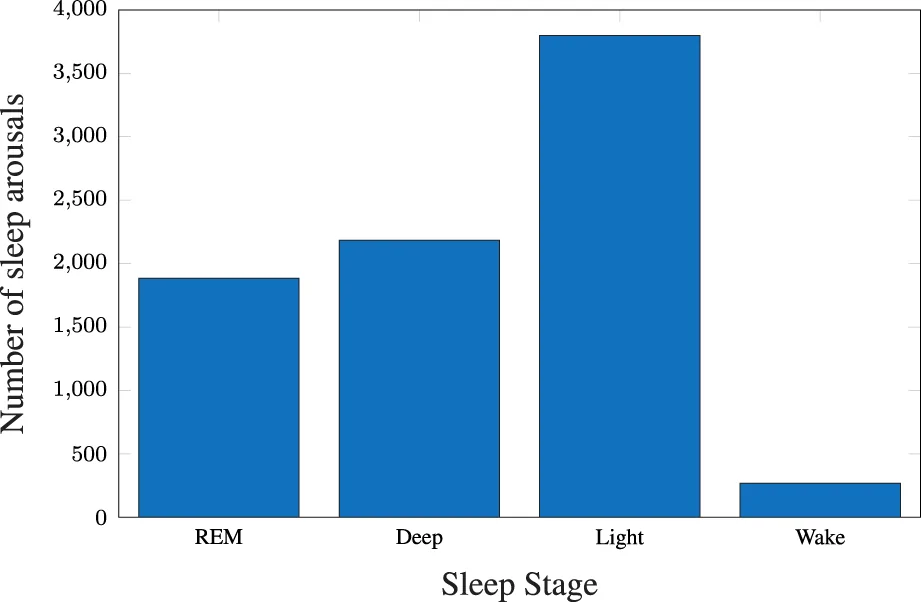
Distribution of sleep stages over the selected arousals. Non-rapid eye movement stages 1 and 2 have been combined to light sleep stage, and non-rapid eye movement stages 3 and 4 have been combined to deep sleep stage. REM, rapid eye movement.

### 2.2 Previous methods of information transfer and limitations

TE has been defined as a metric that quantifies directed predictive information from a source process X to a destination process Y (Schreiber, 2000). Both systems, source and destination, can be characterized by numeric time series. In a TE calculation X→Y , the central question is whether the past of X improves the prediction of the current or future state of Y beyond the information that is already contained in the past of Y (Schreiber, 2000; Bossomaier et al., 2016; Wibral et al., 2013). In information-theoretic terms, TE can be written as the conditional mutual information between the destination state and the source history, conditioned on the destination history (Schreiber, 2000; Wibral et al., 2013; Lizier, 2013).

TE is closely related to the concept of Wiener–Granger causality (Granger, 1969). For Gaussian-distributed variables, TE and Wiener–Granger causality are equivalent (Barnett et al., 2009; Wibral et al., 2013). However, TE is not restricted to linear autoregressive dependencies. Directed information is another information-theoretic framework for directional dependence in time-ordered systems and is especially relevant when feedback is present (Massey, 1990). Massey (1990) emphasized that mutual information has no inherent directivity, whereas directed information can describe directed information flow in feedback systems. In this work, we used TE because it directly describes directed predictive information between physiological feature time series and because the investigated embedding strategies are implemented in the Java Information Dynamics Toolkit (JIDT) (Lizier, 2014).

It should be noted that TE for continuous variables is only an estimate. It is influenced by the distribution of the variables, estimator bias and variance, and the chosen density estimator (Bossomaier et al., 2016; Kraskov et al., 2004). In real-world data, several additional factors affect TE estimation, such as noise, measurement errors, resolution, stationarity, sampling rate, and the limited number of samples (Bossomaier et al., 2016; Kantz and Schreiber, 2003; Müller et al., 2016). These factors are especially relevant for physiological feature time series, because they can be noisy, nonstationary, and influenced by several regulatory mechanisms at the same time.

The estimation of TE also necessitates the definition of the information horizon, which is referred to as the embedding for TE estimation. This problem is closely related to delay-coordinate reconstruction in nonlinear time-series analysis, where a state-space representation is reconstructed from delayed observations of a measured signal (Kantz and Schreiber, 2003). The embedding parameters include the history H and tau τ for the source and destination and the delay d between them (Bossomaier et al., 2016; Wibral et al., 2013). H describes the number of past samples that are included in the embedded state vector. τ describes the distance between these samples. The delay d describes the temporal distance between the source and destination state, as illustrated in Figure 2. Thus, for a TE calculation X→Y , the embedding defines how much source and destination history is included in the calculation and at which temporal spacing.

**FIGURE 2:**
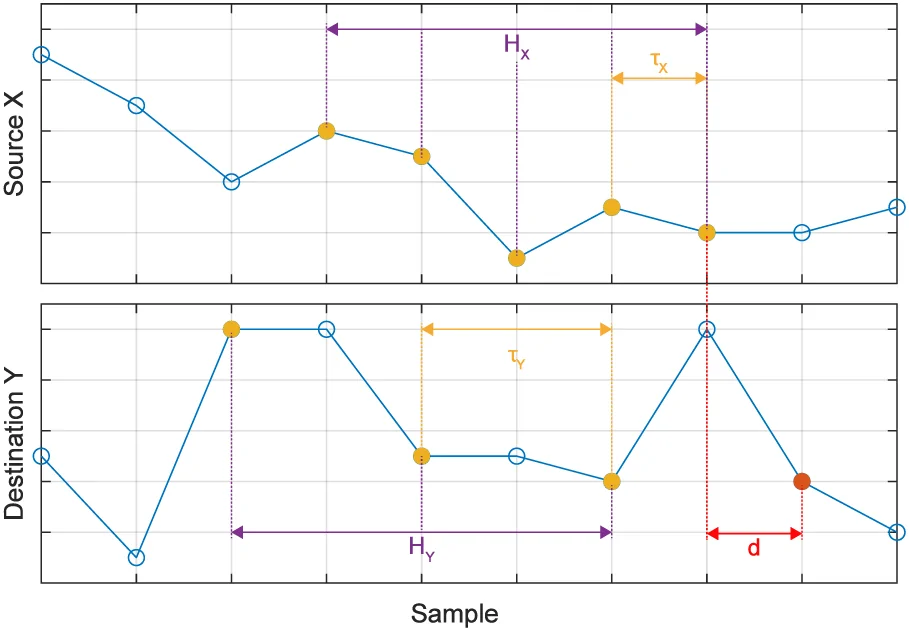
Embedding for TE calculation from source X to destination Y. In this example, the history H (magenta) for source X contains five samples, and for destination Y, it contains three samples. The tau τ (orange) for X is one sample, and for Y, it is two samples. The delay d (red) is one sample.

Embedding can be further divided into uniform and non-uniform embedding. In uniform embedding, the distance between samples is homogeneous, and the past-state vector is defined by fixed values of H and τ . In non-uniform embedding, lagged components are selected individually and may, therefore, be irregularly spaced in time (Faes et al., 2012). Non-uniform embedding has been proposed for cardiovascular and cardiorespiratory variability analysis because it can reduce the dimensionality of conditioning vectors while retaining informative lagged components (Faes et al., 2012). In this work, only uniform embedding was used to ensure a consistent parameter space across all sleep stages, source–destination combinations, and compared embedding strategies.

Interaction-delay estimation is a specific part of the embedding problem. Wibral et al. (2013) demonstrated that TE can be extended to scan source–destination delays while retaining the conditioning on the destination’s past state. This conditioning is important because the destination’s self-predictive information must be adequately represented in the conditioning set; otherwise, information stored in the destination’s own past can be falsely attributed to the source (Wibral et al., 2013). Delay scanning is, therefore, a relevant approach when the true interaction delay is unknown.

Information-based embedding selection has also been proposed for forecasting. Garland et al. (2016) selected delay-coordinate reconstruction parameters by maximizing the information shared between reconstructed states and future states. This approach is related to our work because both use information-theoretic criteria for parameter selection. However, Garland et al. (2016) optimized single-process reconstruction for forecasting, whereas our *max. TE* strategy optimizes source–destination embeddings for directed physiological interaction analysis.

The need to define an information horizon for calculating information transfer raises the question of the optimal method. The *Ragwitz optimization technique (Ragwitz criterion)* is a strategy based on the criteria defined by Ragwitz and Kantz (2002). It aims to minimize the prediction error of the next value in a time series by setting the optimal history length, embedding length, or embedding delay. However, the *Ragwitz criterion* optimizes the prediction of a single time series and does not directly optimize directed source–destination TE (Ragwitz and Kantz, 2002; Kantz and Schreiber, 2003). AIS quantifies information stored in a process’s own past (Lizier, 2013). Both *maximum bias-corrected AIS (Max. Corr. AIS)* and *maximum bias-corrected AIS at maximized TE (Max. Corr. AIS + TE)* strategies sequentially increase the amount of history as long as the additional history provides more information about the next value of a time series than the bias introduced by the higher dimensionality. Max. Corr. AIS + TE subsequently maximizes the bias-corrected TE over source embeddings while accounting only for the information storage of the source. Thus, AIS-based strategies optimize information storage, whereas TE quantifies directed predictive information transfer. An AIS-optimal embedding is therefore not necessarily TE-optimal for a specific source–destination pair (Lizier, 2013). In addition, event-wise optimization in short and noisy physiological windows can lead to unstable parameter choices. This motivated the development of the group-level *max. TE* strategy used in this work.

All investigated comparator strategies were implemented in the *JIDT* toolbox (Lizier, 2014). Since TE estimation can be affected by estimator bias, finite sample size, autocorrelation, and measurement noise, surrogate analyses were used to test whether the observed TE values exceed those expected under a defined null model (Kantz and Schreiber, 2003; Schreiber and Schmitz, 1996). The operational details of the iterative amplitude-adjusted Fourier transform (IAAFT) and time-reversal surrogate validation are described in Section 2.5.

### 2.3 Optimized transfer entropy calculation

In this work, we introduce a novel optimization strategy named *max. TE* and compare it with three established JIDT auto-embedding strategies: *Max. Corr. AIS*, *Max. Corr. AIS + TE*, and the *Ragwitz criterion* (Lizier, 2014; Ragwitz and Kantz, 2002).

With *max. TE*, we address the problem that TE estimation is sensitive to noise, finite sample size, and the selected embedding. *Max. TE* is a more robust approach to embedding for our physiological application because it selects the final embedding parameters based on repeated and comparable arousal events rather than optimizing each event completely independently. Similar events are defined as arousals belonging to the same sleep stage and the same source–destination feature combination. The objective of *max. TE* is not to produce an unbiased absolute TE estimator but to provide stable group-level embeddings for TE calculation in physiological arousal data. Methods such as symbolic transfer entropy can be more robust to noise, but symbolization removes information from the input signal (Hohmuth et al., 2023). Therefore, continuous-valued TE was chosen.

The proposed *max. TE* strategy differs from previous delay-scanning and reconstruction–optimization approaches in its optimization target and level of application. Wibral et al. (2013) scanned source–destination delays to identify information-transfer delays while conditioning on the immediately previous target state. Garland et al. (2016) selected delay-coordinate parameters by maximizing information shared between reconstructed states and future states for forecasting. In contrast, *max. TE* searches over source history, source τ , destination history, destination τ , and source–destination delay to select embeddings that yield stable group-level TE estimates for each sleep stage and source–destination feature pair. Thus, *max. TE* is not intended to estimate subject-specific physiological delays, but to provide comparable embeddings for the physiological group-level analysis.

*Max. TE* optimization is implemented via a grid search across all embedding parameters, specifically the history H and τ of the source and destination time series and the source–destination delay d , within predefined ranges. Embedding parameters were varied from 1 to 9 with a step size of 1 for source history $H_{x}$ , source spacing $\tau_{x}$ , destination history $H_{y}$ , destination spacing $\tau_{y}$ , and source–destination delay d . It should be noted that the embedding parameter grid (1–9 samples, corresponding to 1–9 s at 1 Hz parameter sampling rate) was applied uniformly to all source–destination combinations. The grid was not designed to represent sympathetic or parasympathetic timescales at the level of the raw ECG or EEG, because the physiological effects of both branches of the ANS are already represented by the derived vital parameters themselves (e.g., HF for PNS activity and LF and QT-interval parameters for SNS activity), as summarized in Table 2. The embedding grid therefore operates on the level of directed predictive information transfer between these physiologically interpreted parameter time series, not on the level of the underlying raw signals.

In this study, we applied *max. TE* on 400 selected arousals to quantify information transfer during sleep. The development workflow is shown in Figure 3. The development dataset was balanced across sleep stages, with 100 arousals each for wake, light sleep, deep sleep, and REM sleep. For each subset of the development dataset according to sleep stage, the embedding parameters were optimized. The sample size of 400 arousals in the development dataset was selected based on the required computation time and was not based on physiological considerations.

**FIGURE 3:**
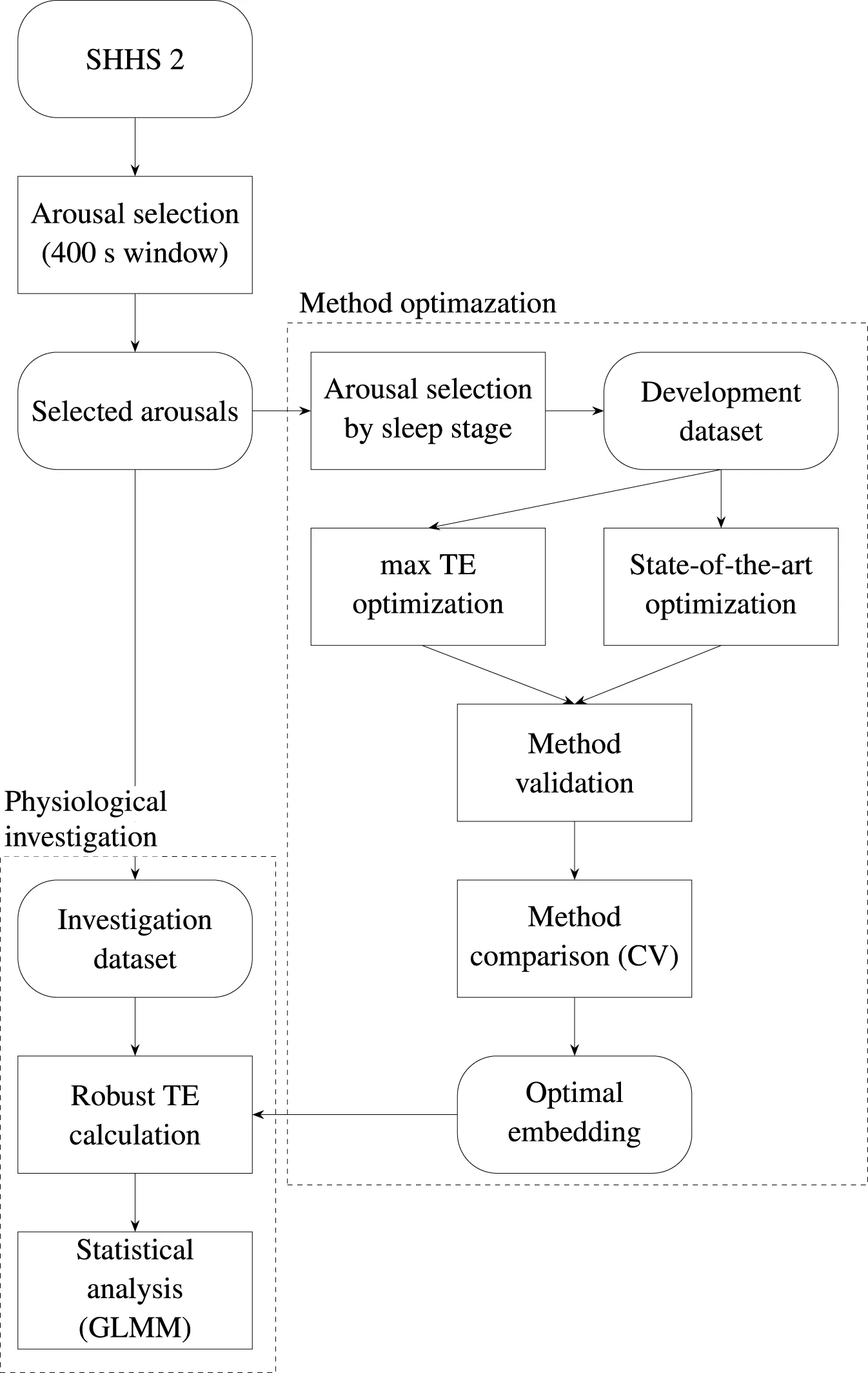
Flowchart of the arousal selection, development, and analysis workflow, including method optimization and physiological investigation. Max. TE, maximum TE optimization; CV, coefficient of variation; GLMM, generalized linear mixed model.

For TE estimation on the selected arousals, the final embedding parameters were chosen based on the most frequently occurring embedding parameters in the development dataset. Arousals that were part of the development dataset were not included in the physiological evaluation. The embedding parameters were specific to the sleep stage and the source–destination parameter combination.

Because *max. TE* selects the largest estimated TE over a grid of candidate embeddings, the procedure can introduce selection-induced upward bias. The potential bias and variance of the estimator were evaluated separately in the simulation benchmark with known ground-truth TE.

### 2.4 Analysis workflow

The analysis workflow is structured to separate method optimization from physiological investigation. Specifically, the development dataset is formed to optimize the novel *max. TE* method and compare its robustness against established JIDT auto-embedding strategies. All strategies were optimized and compared on the same 400 arousals in the development dataset, as shown in Figure 3. The comparison between optimization strategies was performed using the coefficient of variation (CV) of the estimated TE, given that the embedding strategy is expected to robustly estimate information transfer for a given sleep stage and source–destination combination. The CV was calculated as the ratio of the standard deviation to the mean of the TE value within a sleep stage of the development dataset. The investigated strategies were compared based on the average CV across the source–destination combinations and all sleep stages.

A low CV indicates that TE estimates vary less across arousals within the same sleep-stage and source–destination combination. Therefore, CV was interpreted as an empirical stability metric and not as a direct measure of estimator accuracy. In this work, robustness was defined as the ability of an embedding strategy to provide stable and physiologically reliable TE estimates across repeated arousals, sleep stages, and source–destination combinations. We evaluated robustness using four complementary criteria: (i) empirical stability, quantified by CV in the development dataset; (ii) physiological surrogate reliability, quantified by source-only IAAFT surrogate testing and source-time-reversal testing; (iii) source–destination reliability, quantified by the proportion of feature pairs passing the IAAFT surrogate test; and (iv) delay-recovery robustness in simulated systems with known coupling delay. Estimator accuracy was evaluated separately using simulation-derived bias, absolute bias, and root mean square error (RMSE).

For each sleep stage and each combination of source and destination parameters, there is an optimal embedding for TE calculation (see Section 2.3). The final embedding parameters were chosen based on the development dataset and were specific to the sleep stage and the source–destination parameter combination.

The physiological investigation was performed after the optimization of the embedding parameters. Subsequently, the remaining selected arousals were utilized for the application of the methods and the investigation of physiological influencing factors. Arousals that were part of the development dataset were not taken into account for the physiological investigation. Therefore, the physiological investigation was performed on the investigation dataset, consisting of 7,736 arousals from 1,945 subjects. The investigated arousals were grouped by subject and sleep stage. TE was then calculated between all pairs of selected parameters of HRV, QTV, and EEG (see Table 2), using the optimized embedding parameters from the development dataset.

TE estimation and surrogate testing in the investigation dataset were implemented using the *JIDT* toolbox (Lizier, 2014). For continuous-valued physiological feature time series, JIDT provides nearest-neighbor estimators, introduced by Kraskov et al. (2004), for information-theoretic quantities (Lizier, 2014). A surrogate analysis using 200 iterations was performed to validate the calculated TE values. Negative TE values were discarded because TE is defined as non-negative, but negative estimates can occur due to finite-sample effects and probability-density estimation. In addition, all TE values for which the surrogate test failed at a significance threshold of 5% were discarded from further evaluation.

For better numerical readability, we multiplied the TE values by a factor of 1,000 and reported them in milli-nats (mnats), where 1 nat corresponds to 1,000 mnats. Thus, we calculated TE values for each embedding and parameter combination. As this work focuses on information transfer between organ systems, TE values were averaged according to their assignment to SNS, PNS, PNS + SNS, and CNS within each arousal. AHI and arousal index were grouped according to severity. AHI is binned as recommended by the literature into normal (AHI <5 ), mild ( 5≤ AHI <15 ), moderate ( 15≤ AHI <30 ), and severe (AHI ≥30 ) (Ruehland et al., 2009). Arousal index was binned into normal (arousal index <15 ), mild ( 15≤ arousal index <20 ), and significant (arousal index ≥20 ), based on the distribution of the arousal index across all subjects.

### 2.5 Development-set surrogate validation of max. TE

To assess whether the selected *max. TE* embeddings reflected temporally specific source–destination structure rather than univariate signal properties alone, we performed a surrogate validation in the development dataset. Surrogate data are artificial time series generated under a defined null hypothesis and are used to test whether a statistic calculated from the original data exceeds values expected under that null model (Kantz and Schreiber, 2003). In this study, we used IAAFT surrogates. The IAAFT procedure iteratively constructs surrogate time series that preserve the empirical amplitude distribution and approximately preserve the power spectrum, and therefore the autocorrelation structure, of the original signal, while disrupting the original temporal arrangement of the samples (Schreiber and Schmitz, 1996). Schreiber and Schmitz (1996) introduced this iterative approach because simpler amplitude-adjusted Fourier transform surrogates can exhibit finite-sample spectral bias and lead to false rejections of the null hypothesis.

For each development arousal and source–destination feature pair, 200 source-only IAAFT surrogates were generated while the destination signal was kept unchanged. This source-only design preserves the destination dynamics and tests whether the original source time ordering contributes information to the destination beyond the source signal’s own amplitude distribution and autocorrelation structure. For each surrogate realization, TE was recomputed using the embedding selected from the original development-set *max. TE* calculation.

As a complementary temporal-order control, we also used a source-time-reversal surrogate. For this surrogate, the source signal was reversed in time, while the destination signal was kept unchanged. This preserves the source values and distribution but reverses the temporal order of the source relative to the destination. Time-reversal surrogate analyses have been used in physiological interaction studies to test whether an apparent interaction metric persists after the original temporal ordering between two signals has been disrupted (Wessel et al., 2020). We quantified the proportion of source–destination pairs for which the observed TE exceeded the corresponding source-time-reversal TE.

For the IAAFT analysis, a one-sided empirical surrogate *p*-value was calculated as

$$p=\frac{1+\sum_{b=1}^{B}1\left(TE_{b}^{\mathrm{surr}}\geq TE^{\mathrm{obs}}\right)}{B+1},$$

where $TE^{\mathrm{obs}}$ is the observed TE, $TE_{b}^{\mathrm{surr}}$ is the TE obtained from the b th surrogate, and B=200 is the number of IAAFT surrogate realizations. TE was considered significant if p<0.05 .

The surrogate analysis was performed at the embedding selected from the original max. TE optimization. Therefore, this analysis evaluates whether the selected embeddings yield TE values exceeding structured surrogate expectations. However, because the complete max. TE grid search was not repeated for every surrogate realization, this analysis does not fully remove the selection-induced upward bias that may arise from maximizing TE over the embedding grid.

In addition to the sleep-stage-level surrogate summary, we performed a source–destination-level reliability screening. A source–destination pair was classified as always failing if it had at least 50 valid IAAFT tests and never exceeded the IAAFT surrogate null. A pair was classified as low reliability if its IAAFT pass rate was less than or equal to 0.10. The same criteria were applied within each sleep stage, requiring at least 20 valid tests. This screening was used as a diagnostic reliability analysis of source–destination combinations.

### 2.6 Simulation benchmark of embedding strategies

To evaluate the estimator accuracy independently of physiological data, we benchmarked *max. TE* and the three JIDT auto-embedding strategies on simulated linear Gaussian systems with known coupling structure and analytically computed ground-truth TE. This simulation benchmark complements the surrogate validation. The surrogate validation tests whether physiological *max. TE* values exceed structured null expectations, whereas the simulation benchmark quantifies bias, RMSE, delay recovery, and runtime under known ground truth.

The simulated system was defined as

$$X_{t}=a_{x}X_{t-1}+\epsilon_{t}^{X},$$

$$Y_{t}=a_{y}Y_{t-1}+cX_{t-d}+\epsilon_{t}^{Y},$$

where d is the true source–destination delay, c is the coupling strength, and $\epsilon_{t}^{X}$ and $\epsilon_{t}^{Y}$ are Gaussian noise terms. We varied sample length (N=300,500) , true delay (d=1,3,5) , and coupling strength (c=0,0.15,0.35,0.60) . For each scenario, 20 Monte Carlo repetitions were generated.

Estimator accuracy was quantified using bias, absolute bias, and RMSE. Bias was defined as

$$Bias=\hat{TE}-TE_{\mathrm{true}},$$

where $\hat{TE}$ is the estimated TE and $TE_{\mathrm{true}}$ is the analytically computed ground-truth TE of the simulated system. Positive bias indicates systematic overestimation, whereas negative bias indicates systematic underestimation. RMSE summarizes the average magnitude of the estimation error across Monte Carlo repetitions and was calculated as

$$RMSE=\sqrt{\frac{1}{N_{\mathrm{rep}}}\overset{N_{\mathrm{rep}}}{\underset{i=1}{\sum }}{\left({\hat{TE}}_{i}-TE_{\mathrm{true}}\right)}^{2}},$$

where $N_{\mathrm{rep}}$ is the number of Monte Carlo repetitions. Lower RMSE indicates higher accuracy with respect to the known ground-truth TE.

Robustness was evaluated separately from accuracy. We used the coefficient of variation (CV) of the TE estimates across Monte Carlo repetitions as a stability metric, and the delay recovery rate as a measure of whether the method identified the true source–destination delay. Thus, RMSE and bias quantified estimation accuracy, whereas CV and delay recovery quantified the robustness of the embedding strategy.

For the JIDT auto-embedding strategies, the following methods were evaluated: *Max. Corr. AIS*, *Max. Corr. AIS + TE*, and the *Ragwitz criterion*. The auto-embedding delay range was 1–9, with maximum embedding and τ search values of 15. Runtime was measured as wall-clock time per simulated source–destination pair.

### 2.7 Statistical analysis

A generalized linear mixed model (GLMM) was employed to investigate the physiological effects of sleep stage, CVD, sex, age, AHI, and arousal index on information transfer between organ systems. The analysis was conducted using the GENLINMIXED procedure in *IBM SPSS (IBM Corp., released 2024; Version 30, Armonk, NY)*. The fixed effects entered into the model were sleep stage, age, sex, CVD, parameter combination (source to destination), binned AHI, and binned arousal index. To account for the non-independence of observations arising from the hierarchical data structure, a nested random-effects structure was specified. Random intercepts were included at three levels: the unique identifier of each arousal, the identifier of each subject, and the combination of the sleep-stage arousal identifier and subject identifier. This allows for modeling subject-specific and condition-specific deviations from the global intercept. The covariance structure for these random effects was modeled using the variance component type. The residual covariance structure was set to diagonal, which assumes that the errors are uncorrelated but allows them to have different variances at each level of the repeated measures for the parameter combination. Model parameters were estimated via maximum likelihood. To ensure the robustness of our statistical inferences against potential misspecification of the covariance structure, robust standard errors (sandwich estimator) were calculated for the fixed-effects estimates. For significant effects, estimated marginal means were calculated, and pairwise comparisons were performed. The resulting *p*-values were corrected using the Bonferroni–Holm method. Statistical significance was set at α=0.05 .

## 3 Results

### 3.1 Method optimization and comparison to the state-of-the-art

To determine the most robust embedding parameters for quantifying information transfer between the ANS and CNS, we first optimized the embedding parameters through a systematic evaluation of four optimizing strategies, as described in Section 2.4. All subsequent TE calculations were performed on the selected arousals excluded from the development dataset using *max. TE* as the embedding strategy. Table 3 summarizes the embedding parameters and the corresponding CV for each sleep stage in the development dataset. The CV is lower during REM sleep (0.29) and higher during light and deep sleep (0.33). The optimal embeddings for *max. TE* are illustrated for each source–destination combination and sleep stage and can be found in the Supplementary Material. For the specified embedding parameters *destination history* $H_{y}$ , *destination tau* $\tau_{y}$ , *source history* $H_{x}$ , *source tau* $\tau_{x}$ , and *delay* d , the optimal embedding parameters across organ systems (ANS and CNS) are 1 across all embedding parameters, except the pNN50 parameter, for which the embedding parameters are distributed between 3 and 7. The optimal embedding parameters within the ANS or CNS are distributed across the range of the grid search. Compared with the three state-of-the-art embedding strategies, *max. TE* yielded the lowest empirical CV in the physiological development dataset, indicating the most stable TE estimates under the predefined CV criterion. The mean CV was 0.32 for *max. TE*, compared with 0.39 for Max. Corr. AIS + TE, 0.48 for Max. Corr. AIS, and 1.20 for the *Ragwitz criterion*. Thus, *max. TE* achieved a 17.9% lower CV than the second-most stable strategy, Max. Corr. AIS + TE, as illustrated in Table 4. The robustness interpretation was supported by the development-set surrogate validation. Across all 400 development arousals, observed *max. TE* exceeded source-only IAAFT surrogate TE in every sleep stage. The mean observed TE was 135.9, 136.8, 139.6, and 141.5 mnats for wake, light, deep, and REM sleep, respectively, whereas the mean IAAFT TE was 88.2, 89.2, 91.2, and 93.3 mnats. The mean IAAFT pass rate ranged from 0.807 to 0.817 across sleep stages, and the median empirical IAAFT *p*-value ranged from 0.005 to 0.007. Observed TE also exceeded source-time-reversal TE in 0.825–0.840 of directed pairs across sleep stages. At the source–destination level, no retained feature pair always failed the IAAFT surrogate test. The median IAAFT pass rate across pairs was 0.865, and no pair had an overall pass rate below or equal to 0.10. In the simulation benchmark with known ground-truth TE, *max. TE* showed a different robustness profile than the auto-embedding methods. Robustness and accuracy metrics for the investigated embedding strategies are shown in Table 5. *Max. TE* achieved the highest delay-recovery rate among the tested strategies, with a mean delay recovery of 0.652 compared with 0.560 for Max. Corr. AIS, 0.517 for Max. Corr. AIS + TE, and 0.304 for *Ragwitz*. *Max. TE* also showed low variability across Monte Carlo repetitions, with a mean CV of 0.124, comparable to that of Max. Corr. AIS + TE (0.116) and lower than those of Max. Corr. AIS (0.307) and *Ragwitz* (0.398).

**TABLE 3:** Comparison of embedding parameters across sleep stages for max. TE. History H , spacing τ , and delay d are reported in samples. CV, coefficient of variation.

Sleep stage	Wake	Light	Deep	REM
Destination $H_{y}$	1.80 ± 1.24	1.76 ± 1.24	1.76 ± 1.23	1.84 ± 1.24
Source $H_{x}$	5.34 ± 2.59	5.46 ± 2.60	5.60 ± 2.59	5.45 ± 2.53
Destination $\tau_{y}$	2.31 ± 2.11	2.26 ± 2.07	2.09 ± 1.95	2.02 ± 1.88
Source $\tau_{x}$	6.63 ± 2.58	6.67 ± 2.53	6.68 ± 2.52	6.67 ± 2.51
Delay	4.67 ± 2.96	4.70 ± 2.95	4.63 ± 2.94	4.67 ± 2.93
CV	0.31	0.33	0.33	0.29

**TABLE 4:** Comparison of embedding strategies by the coefficient of variation (CV).

Embedding strategy	CV
Ragwitz criterion	1.20±0.02
Max. Corr. AIS	0.48±0.02
Max. Corr. AIS + TE	0.39±0.06
Max. TE	0.32±0.02

**TABLE 5:** Robustness and accuracy metrics for the investigated embedding strategies: maximum TE optimization (max. TE), maximum bias corrected AIS (Max. Corr. AIS), maximum bias corrected AIS at maximized TE (Max. Corr. AIS + TE), and Ragwitz criterion.

Metric	Max. TE	Max. Corr. AIS	Max. Corr. AIS + TE	Ragwitz
Development CV	0.32	0.48	0.39	1.20
Simulation CV	0.124	0.307	0.116	0.398
Delay recovery	0.652	0.560	0.517	0.304
RMSE (mnats)	64.4	30.6	66.9	62.2
Mean bias (mnats)	61.5	14.0	64.2	−20.5
Runtime (s)	33.2	23.1	29.5	14.6

Development coefficient of variation (CV) was calculated in the physiological development dataset. Simulation CV, delay recovery, root mean square error (RMSE), bias, and runtime were calculated in the linear Gaussian benchmark with known ground-truth transfer entropy (TE). Lower CV indicates higher empirical stability; higher delay recovery indicates better identification of the true interaction delay. RMSE and bias quantify estimator accuracy and are reported separately from robustness.

We tested all optimization strategies against each other for statistically significant differences using a two-sided Wilcoxon signed-rank test. After applying a *p*-value correction using the Bonferroni-Holm method, it was found that each strategy showed significant differences from all other strategies for every sleep stage (p<0.05) . For the investigation dataset, 9.2% of TE calculations failed the surrogate test and were, therefore, discarded for further calculations.

### 3.2 Physiological investigation

The calculated GLMM (*p* < 0.001) reflects the effects of sleep stage, age, sex, CVD diagnosis, AHI, arousal index, and the source–destination parameter combination as fixed effects on the TE value. The results of this analysis are presented in Table 6. The investigation revealed a statistically significant (p<0.05) impact of all fixed effects on the TE value. The F-value indicates which fixed effects contribute most strongly to explaining the variance in the TE value. The source–destination parameter combination shows the largest test statistic (F=3372.27) , identifying it as the dominant predictor. In contrast, the arousal index (F=3.12) and the AHI (F=5.36) yield the smallest F-values and therefore contribute the least. Subsequent analysis of the GLMM model output delineates the isolated effects of the influence factors on the TE value.

**TABLE 6:** Statistical results of fixed effects on the TE value of the generalized linear mixed model (GLMM) output.

Fixed effect	F-value	Corrected *p*-value
Sleep stage	5.12	0.005
Age	46.16	<0.001
Sex	8.11	0.009
CVD	15.45	<0.001
AHI	5.36	0.004
Arousal index	3.12	0.044
Source–destination combination	3372.27	<0.001

The *p*-values have been corrected using the Bonferroni–Holm method. CVD, cardiovascular diseases; AHI, apnea-hypopnea index.

#### 3.2.1 Influence of sleep stages

The highest information transfer was found during REM sleep, with a TE of 88.2±0.5 mnats, while the lowest information transfer was found during wake, with a TE of 84.0±1.6 mnats. A GLMM *post hoc* analysis of the influence of sleep stages demonstrated that information transfer during wake differed significantly (p<0.05) from that during all other stages (see Figure 4). Significant differences were also found between light and deep sleep (GLMM: p<0.05 ) and between deep and REM sleep (GLMM: p<0.01 ).

**FIGURE 4:**
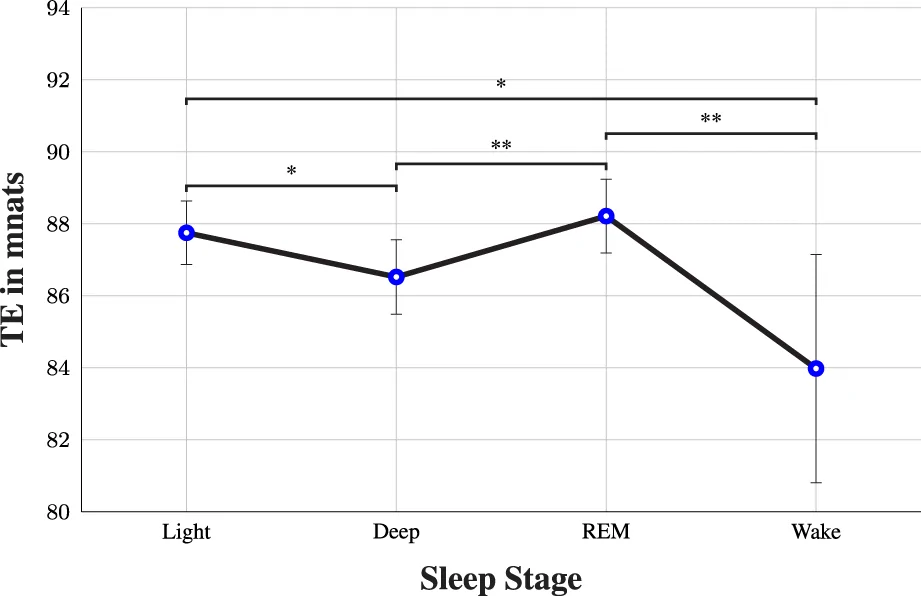
Mean TE between investigated systems: parasympathetic nervous system (PNS), sympathetic nervous system (SNS), PNS + SNS, and central nervous system (CNS) across all sleep arousals during the respective sleep stage. REM: rapid eye movement. Statistical significant differences, *p*-values: *, p<0.05 ; **, p<0.01 .

#### 3.2.2 Influence of cardiovascular disease

We found that subjects with CVD had an overall significantly (GLMM: p<0.001 ) lower TE averaged over all system combinations with a TE of 85.4±0.8 mnats compared to healthy controls with a TE of 87.9±0.5 mnats (see Figure 5).

**FIGURE 5:**
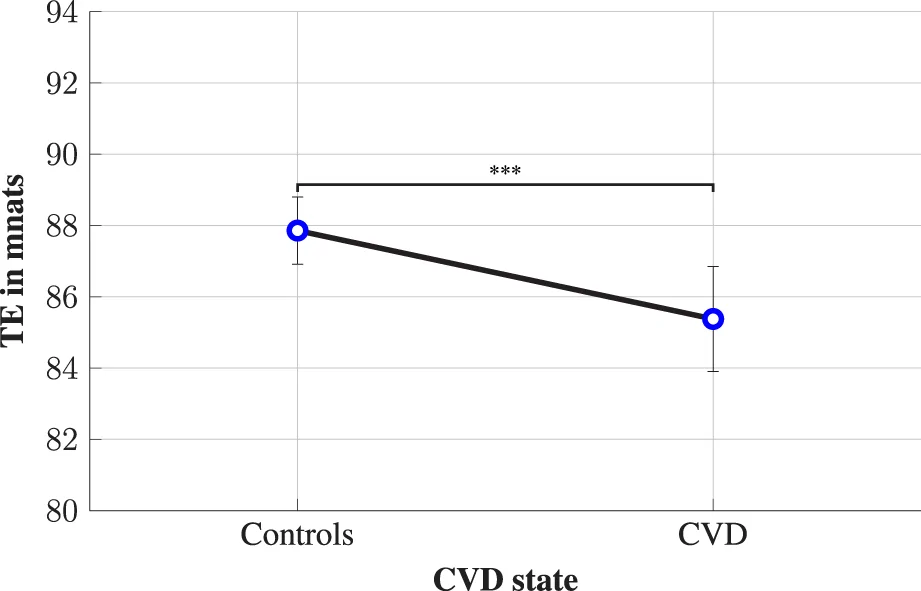
Mean TE between investigated systems: PNS, SNS, PNS + SNS, and CNS for healthy controls and patients with cardiovascular diseases (CVD). Statistical significant differences: ***, p<0.001 .

#### 3.2.3 Interactions between ANS and CNS

Figure 6 illustrates the mean TE among the examined parameter combinations. The lowest information transfer was observed to the PNS as the destination, with the globally lowest TE of 45.2 mnats for information transfer within the PNS. The maximum TE was observed from the SNS to the CNS, reaching 110.3 mnats. In general, the mean TE values from ANS to CNS parameters exceed those from CNS to ANS parameters, with average values of 105.7 mnats and 81.4 mnats, respectively.

**FIGURE 6:**
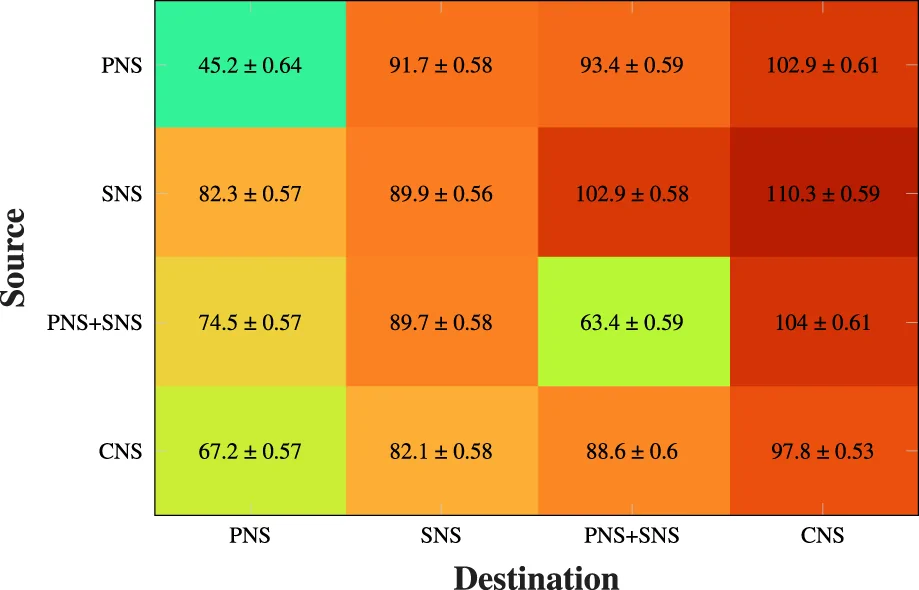
Mean TE between investigated systems, PNS, SNS, PNS + SNS, and CNS.

#### 3.2.4 Demographic influence

We also examined the influence of demographic factors such as age, sex, reported AHI, and arousal index on information transfer. We found a linear trend of decreasing information transfer (slope = −0.1 mnats/yrs, intercept = 98.3 mnats, *R* ^2^ = 0.55, *p*-value < 0.001) in subjects with increasing age. Figure 7 shows the regression of information transfer *versus* subject age. Averaged over all source–destination combinations, we found a significant (GLMM: p<0.001 ) difference in information transfer between both sexes, with a TE for male subjects of 86.0±0.6 mnats compared to 87.2±0.6 mnats for female subjects, illustrated in Figure 8a. No linear trend was found for AHI severity (normal, mild, moderate, and severe) (see Figure 8b) and arousal index severity (normal, mild, and significant) (see Figure 8c). A statistically significant (GLMM: p<0.05 ) difference was identified between the AHI severity categories labeled mild and moderate and between mild and severe. However, no such differences were observed for the normal severity or between mild and moderate. The analysis revealed significant (GLMM: p<0.05 ) differences in the arousal index between normal and significant, and mild and significant, with the lowest TE for significant severity at 85.6±0.7 mnats.

**FIGURE 7:**
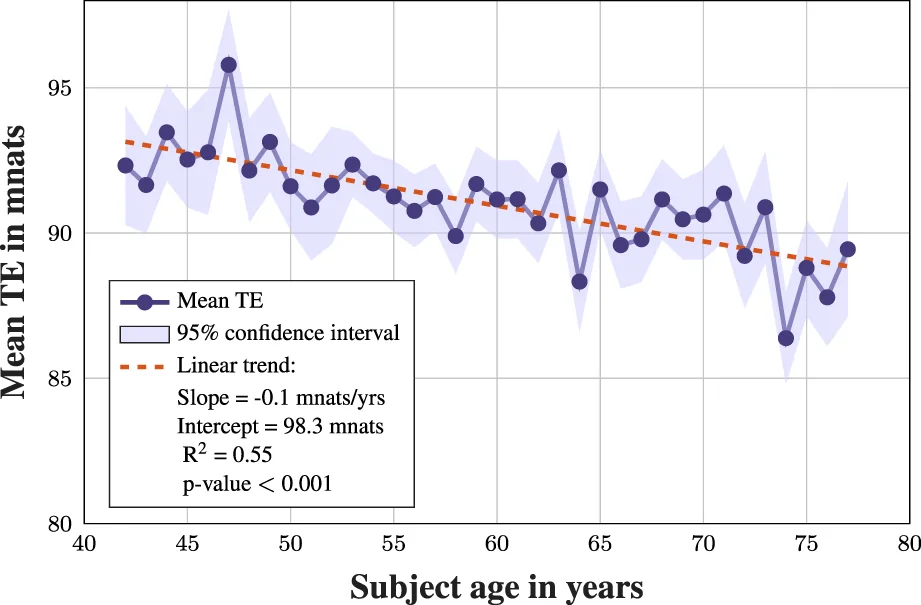
Mean TE and 95% confidence interval over subject age (blue). Figure only included ages with more than 100 subjects (42–77 years). Linear trend of information transfer over subject age (red).

**FIGURE 8:**
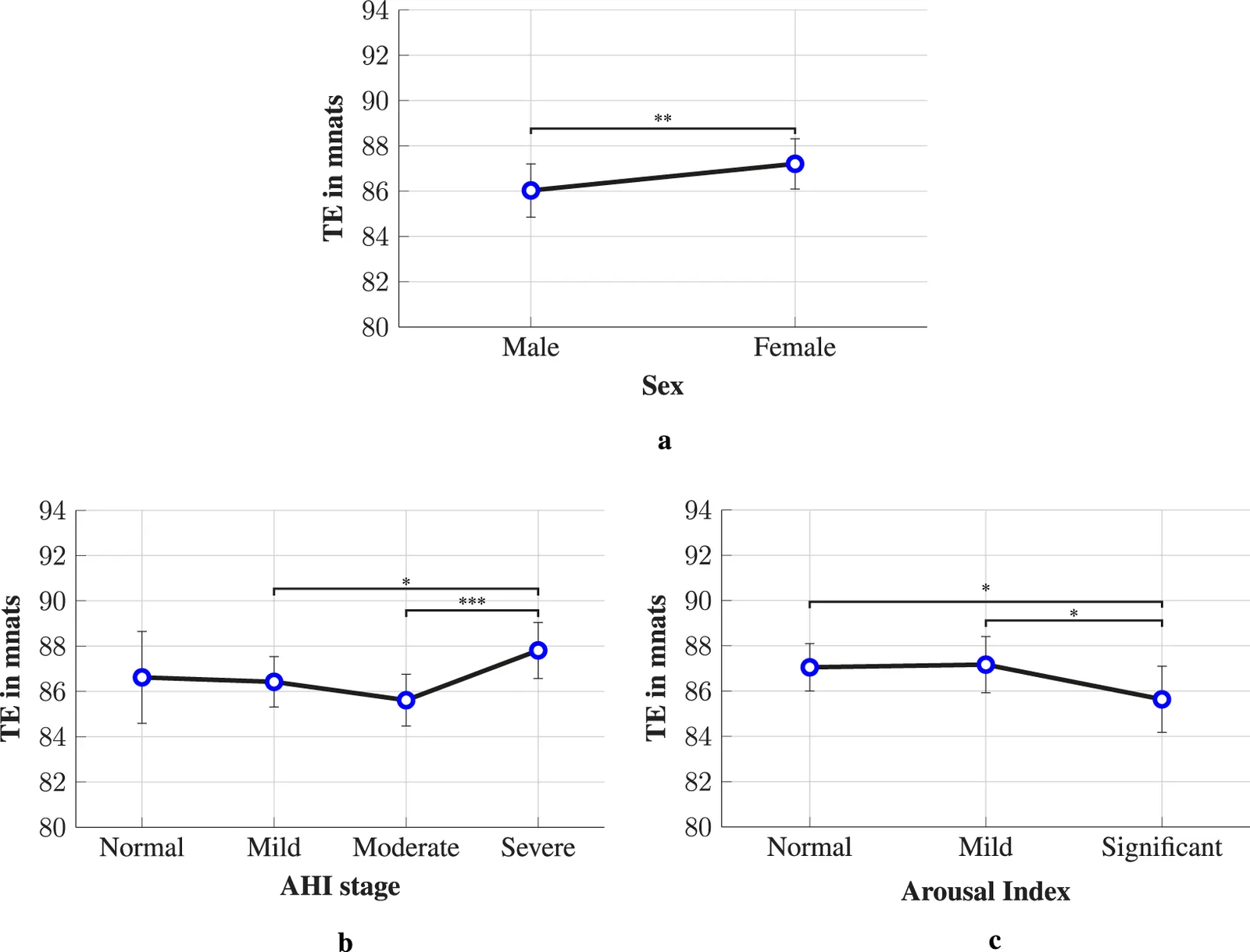
Mean TE of influence factors: **(a)** Sex, **(b)** apnea-hypopnea index (AHI) severity, and **(c)** arousal index severity. Statistical significant differences: *, p<0.05 ; **, p<0.01 ; ***, p<0.001 .

## 4 Discussion

### 4.1 Optimized TE calculation

Various methods have been applied to model information transfer in biological systems (Faes et al., 2014; Faes et al., 2016; Bashan et al., 2012; Bartsch et al., 2014). Since the required *a priori* knowledge is limited to understanding the time domain components of, e.g., regulatory mechanisms such as respiratory sinus arrhythmia or the baroreflex, the necessary embedding of the TE computation is still based on model approaches. We compared four embedding strategies using CV as a stability metric to show how robust these strategies can model information transfer during human sleep. Our proposed method, max. TE, was more robust for the intended physiological group-level application with the lowest CV of 0.32, but it should not be interpreted as the universally most accurate or least biased TE estimator. In the physiological development dataset, *max. TE* yielded the lowest CV, indicating the highest empirical stability across repeated arousals, sleep stages, and source–destination combinations. This stability was complemented by strong surrogate reliability: observed *max. TE* exceeded source-only IAAFT and source-time-reversal surrogate TE across all sleep stages, and no source–destination pair systematically failed the IAAFT surrogate test. Compared with *Max. Corr. AIS + TE*, the second-most stable strategy, *max. TE* reduced the development-set CV by 17.9%. It is not necessarily the case that the embedding parameters for each sleep stage accurately model information transfer, but this strategy supports our hypothesis that each sleep stage represents a unique human state characterized by unique information transfer between all investigated organ systems. This is supported by the GLMM, which revealed a significant effect of sleep stage on information transfer (*p* = 0.005).

For the specified embedding parameters—*destination tau*, *source history*, *source tau*, and *delay*—the parameters across organ systems (ANS and CNS) are set to 1, whereas the within-system combinations (within ANS or CNS) are distributed across the parameter range of the grid search. The pNN50 parameter is the exception, for which the embedding parameters are distributed across the grid range; the reason for this insensitivity remains to be investigated. This distribution of embedding parameters is reflected in the standard deviation of the embedding parameters, which ranges from one to three samples, as shown in Table 3. This phenomenon cannot be attributed to any single cause as it was anticipated that all parameter combinations would fluctuate within the range of the grid search. The underlying reason may be related to the domain differences between EEG- and ECG-derived parameters. The underlying reasons for the pNN50 parameter’s insensitivity to these variations remain to be investigated. The present approach is complementary to recent model-based physiological methods. For example, Rosas et al. (2024) proposed a Bayesian state-space framework in which heart rate is treated as a hidden stochastic process that drives observed heartbeats, yielding posterior distributions rather than point estimates of heart-rate dynamics. Such generative state-space models are well suited for estimating latent autonomic outflow and uncertainty in heart-rate dynamics, whereas the present *max. TE* approach focuses on robust group-level estimation of directed predictive information between derived physiological feature time series. Future work should compare TE-based network measures with generative state-space models, particularly for subject-level physiological assessment.

### 4.2 Sleep stage

With our sleep-stage-specific embedding, we observed the highest mean TE for arousals occurring during REM sleep and the lowest mean TE during wake. Therefore, we conclude that sleep arousals reveal sleep-stage-specific information-transfer dynamics, with REM-related arousals showing the highest directed predictive information transfer in this dataset. The information transfer during wake differed significantly from light and REM. Significant differences were also found between light and deep sleep and between deep and REM sleep. TE is not a sufficient metric to prove that the processes requiring information transfer are similar during both sleep stages, but the amount of information transferred is similar. We observed the same behavior for light and deep sleep, where both sleep stages showed a significant difference to wake and REM sleep, but there was no significant difference in information transfer between the two. This is consistent with the established autonomic regulation during sleep, which is characterized by a shift between parasympathetic dominance during NREM sleep and sympathetic activation during REM sleep (Schmidt et al., 2019; Tobaldini et al., 2013; Gurubhagavatula, 2015). Furthermore, sleep stage has a greater impact on information transfer within the ANS than the interaction between ANS and CNS or within the CNS, which aligns with previous findings that cardio-respiratory coupling forms transition dynamically across sleep stages (Bartsch et al., 2014) and that physiological network interactions are sleep-stage dependent (Krefting et al., 2017). The investigated PNS and SNS parameters are not only influenced by sleep stage; furthermore, the processes during sleep require different information transfer, which can be investigated using our information-theoretic approach.

### 4.3 Cardiovascular disease

Cardiovascular disease inhibits overall information transfer, resulting in a lower mean TE of 85.4 mnats compared to a mean TE of 87.9 mnats in healthy controls. We, therefore, hypothesize that CVD affects the pathways of information transfer between the SNS, PNS, and CNS. More specifically, we found that information transfer within parts of the ANS is inhibited, which is consistent with the literature showing that CVD disrupts regulatory functions within the ANS during sleep (Schmidt et al., 2019), while information transfer between the ANS and CNS increases. We think that the CNS is trying to cope with the disturbed regulatory function of the ANS. Our information-theoretic approach is able to show the disturbance of the regulatory mechanisms within the ANS, as well as the compensation mechanism of the CNS.

The investigation of the transmission of information between the examined organ systems has demonstrated that, following arousals, the transfer of information from the ANS to the CNS is, in general, greater than the reverse, as illustrated in Figure 6. As arousals are defined as spontaneous activations of the CNS (Horner, 1996), it was hypothesized that higher information transfer would be observed within the CNS. These findings suggest that the investigated arousals have an intrinsic source that leads to the transfer of information, enabling the cortex to undergo an awakening reaction.

The subject’s age showed a large effect on information transfer between all combinations of source and destination parameters. With increasing age, information transfer decreases, which is consistent with previous research (Krefting et al., 2017). The decreasing information transfer may not only occur due to age but also due to age-related factors, such as a higher prevalence of diseases. We have not investigated this further as TE is not suitable for individual subject analysis, and only cardiovascular diseases are tagged in the dataset.

### 4.4 Demographic factors

We also examined subject sex and found no significant effect on information transfer. However, the mean TE between male and female subjects was significantly different, with female subjects having a higher TE of 87.2 mnats compared to 86.0 mnats in male subjects. The results are consistent with the findings of earlier research (Krefting et al., 2017). AHI and the arousal index showed medium and large effect sizes, respectively, on information transfer between all examined subsystems. However, a linear regression did not identify a linear correlation between mean TE across all parameter combinations and AHI or the arousal index. Thus, the information-theoretic connection between the two influencing factors must occur at the parameter-combination-specific level.

### 4.5 Regulatory mechanisms

Even though the temporal relationships between regulatory mechanisms such as the baroreflex and respiratory sinus arrhythmia are known, this knowledge cannot be easily translated into an embedding for calculating information transfer. The physiological relevance of TE-based interaction analysis is supported by prior cardiorespiratory work. Schreiber’s original TE study demonstrated bidirectional information transfer between heart-rate and breath-rate recordings in sleep-apnea data, illustrating that TE can reveal directional physiological dependencies not captured by time-delayed mutual information alone (Schreiber, 2000). More recently, Keshmiri et al. (2024) showed that respiration-to-heart-rate TE was informative for cardiorespiratory phenotyping and alcohol-related physiological differences. Although the present study did not include respiration as an explicit source, these findings support the broader use of information-theoretic approaches for quantifying directed physiological interactions. The information transfer responsible for regulatory mechanisms can be spread over a longer period of time. Furthermore, the parameters considered here do not directly reflect the influence of regulatory mechanisms. Rather, the parameters are influenced by a variety of physiological processes. Based on our current knowledge, it is not possible to clearly isolate the influencing factors. Nevertheless, our results indicate that the sum of the processes and regulatory mechanisms contained in the optimized embedding is influenced by sleep stage, sex, age, and CVD, even if these cannot be clearly separated from each other using our method.

### 4.6 Conclusion and further directions

Assessing information transfer is a challenging task, especially in biological systems and real-world data. The dynamics underlying information transfer are complex, and often more than one process influences the vital parameters investigated in this work. Furthermore, real-world data are biased by measurement errors and noise. We addressed those issues and introduced a new embedding method, named *max. TE*, to overcome the embedding problems. Our work compared *max. TE* with three state-of-the-art embedding strategies and showed that *max. TE* was more robust for the intended physiological group-level application. This conclusion is supported by lower empirical CV in the development dataset, strong development-set surrogate reliability, absence of systematically failing source–destination pairs, and the highest delay-recovery rate in the simulation benchmark. Simulation results indicated a positive bias and a higher RMSE compared to Max. Corr. AIS, while *max. TE* exhibited the highest computational cost, measured as runtime on the same machine, as summarized in Table 5. Therefore, *max. TE* should be understood as a robust group-level embedding strategy for physiological information-transfer analysis, not as an unbiased estimator of absolute TE. We have selected sleep arousal as a natural stimulus, given its capacity to elicit an autonomic response. The present study aims to evaluate information transfer following sleep arousal, with the objective of investigating the influence of CVD, age, sex, AHI, and arousal index. With our embedding method, we calculated the optimal embedding for the sleep stages: wake, light, deep, and REM. A GLMM revealed significant differences in information transfer across the sleep stages. Our results also indicate that subjects with a CVD diagnosis show a lower information transfer compared to healthy individuals, leading to the hypothesis that CVD inhibits information transfer within the physiological network between the PNS, SNS, and CNS. While we can show the influence of sleep stage and CVD on information transfer, further research is necessary to identify the information pathways disrupted by CVD and investigate how restoring those pathways can improve the quality of life of patients. In this study, we supported the validity of our new methodology by providing results consistent with existing literature. While this investigation aligns with the literature and indicates the generalizability of our approach, further studies are needed to confirm that the observed relationships hold in other settings and cohorts. Once this has been established, the methodology can be used to address additional scientific questions and open new fields of application, ultimately enabling its use in real-world scenarios.

## Data Availability

The original contributions presented in the study are included in the article/Supplementary Material, further inquiries can be directed to the corresponding author.
