# Supplementary material for: Robust information transfer calculation reveals dynamic networks during sleep arousal
Source: Front Netw Physiol. 2026 Sep 17;6:1930617. doi: 10.3389/fnetp.2026.1930617 (PMC13627025; doi:10.3389/fnetp.2026.1930617)
Supplement: Supplementary file 1 [file Supplementaryfile1.pdf]

frontiers

Supplementary Material

1 SUPPLEMENTARY DATA

This is the supplementary material for 'Robust Information Transfer Calculation Reveals Dynamic Networks during Sleep Arousal'. It contains the optimal embedding parameters calculated using the maximum TE optimization technique introduced in the referenced main work. The embedding parameters are shown for each of the investigated sleep stages: wake, light sleep, deep sleep and REM sleep. The embedding domains are destination history, destination τ, source history and source τ, as well as delay. Optimal embedding parameters were calculated for every combination of source and destination parameters.

2 SUPPLEMENTARY FIGURES

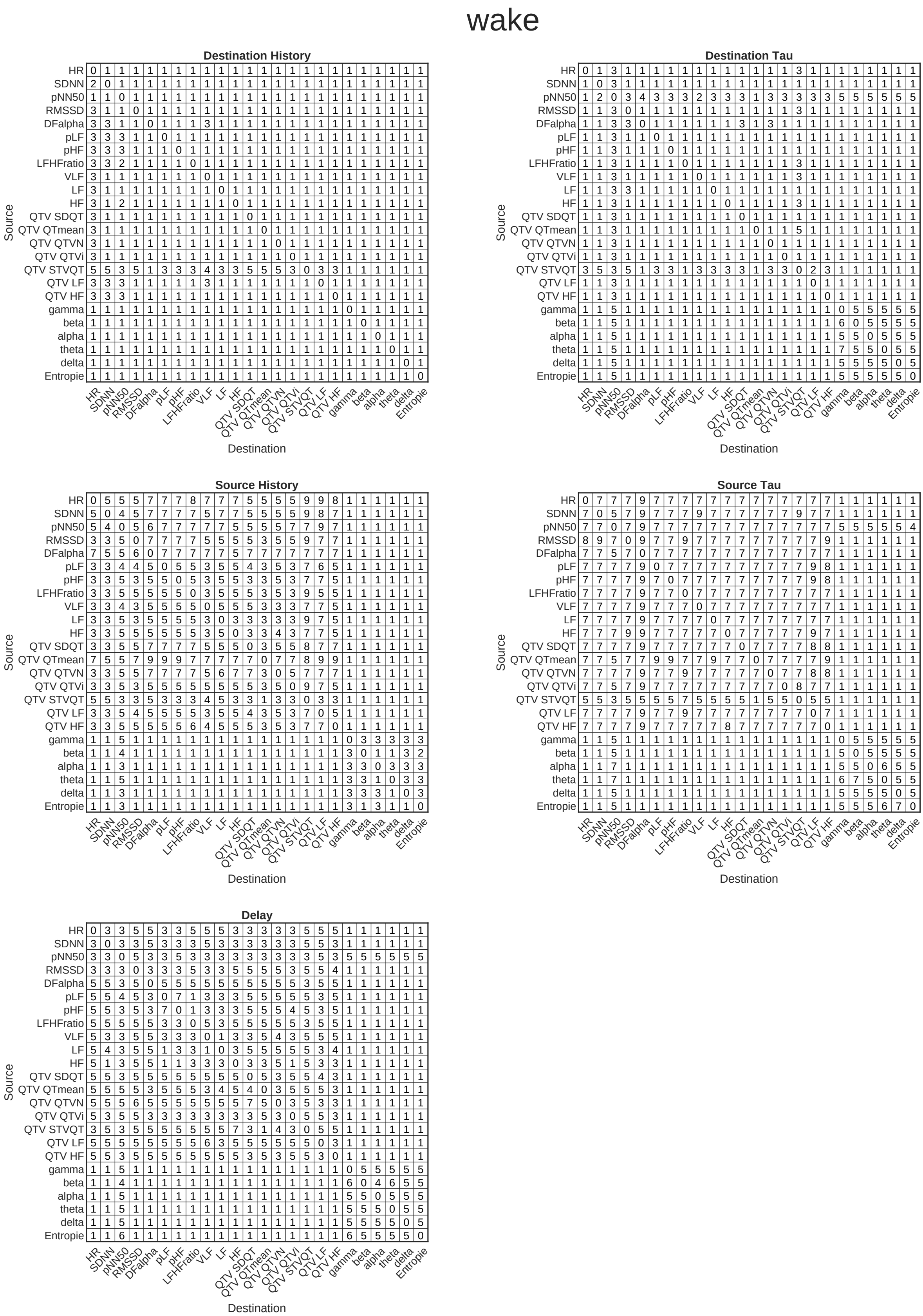

Figure S1. Embedding parameters during sleep stage: wake. Individual calculated for every source and destination parameter combination. Embedding parameters consists of history length and tau τ for source and destination separately and the delay between source and destination.

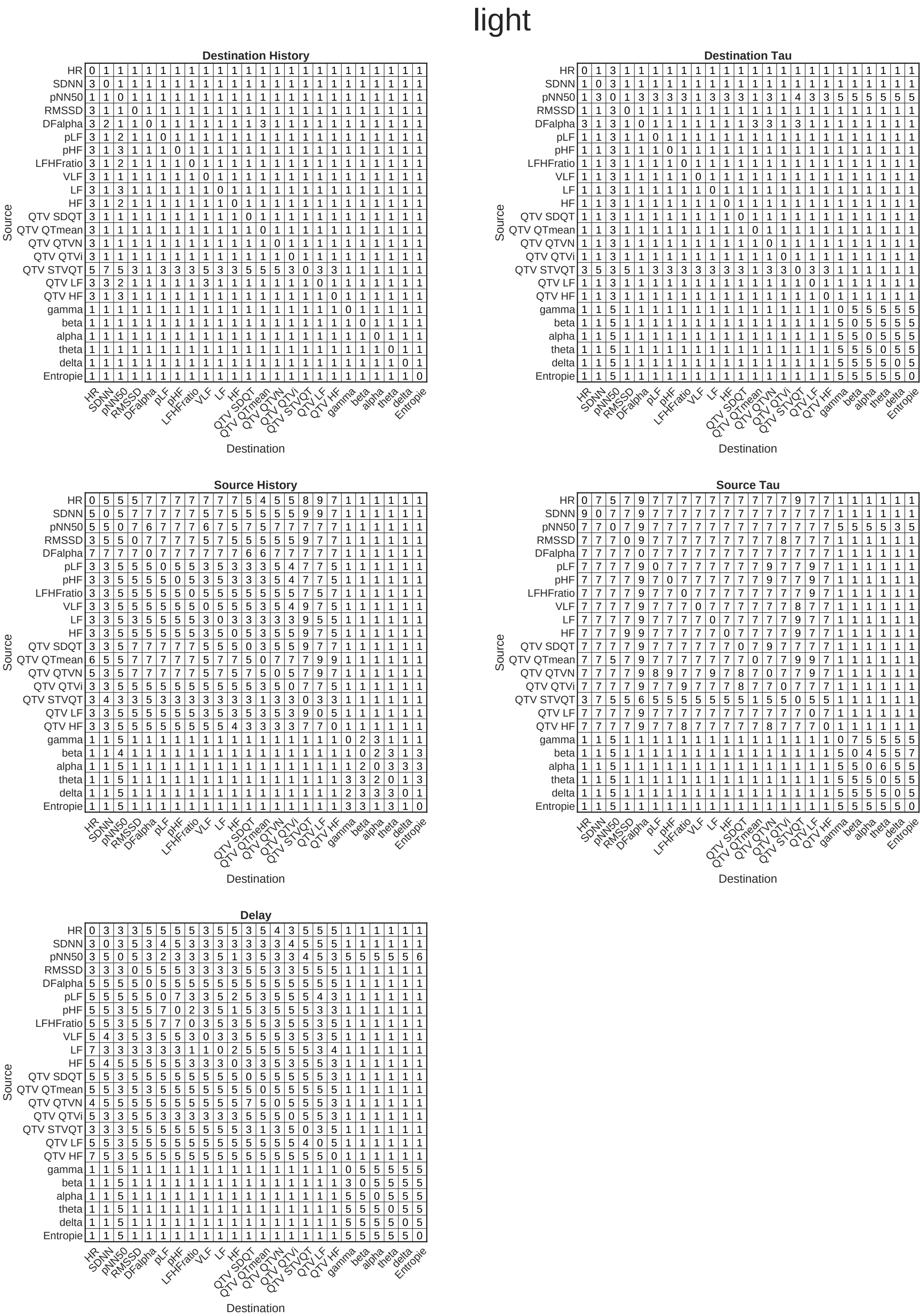

Figure S2. Embedding parameters during sleep stage: light. Individual calculated for every source and destination parameter combination. Embedding parameters consists of history length and tau τ for source and destination separately and the delay between source and destination.

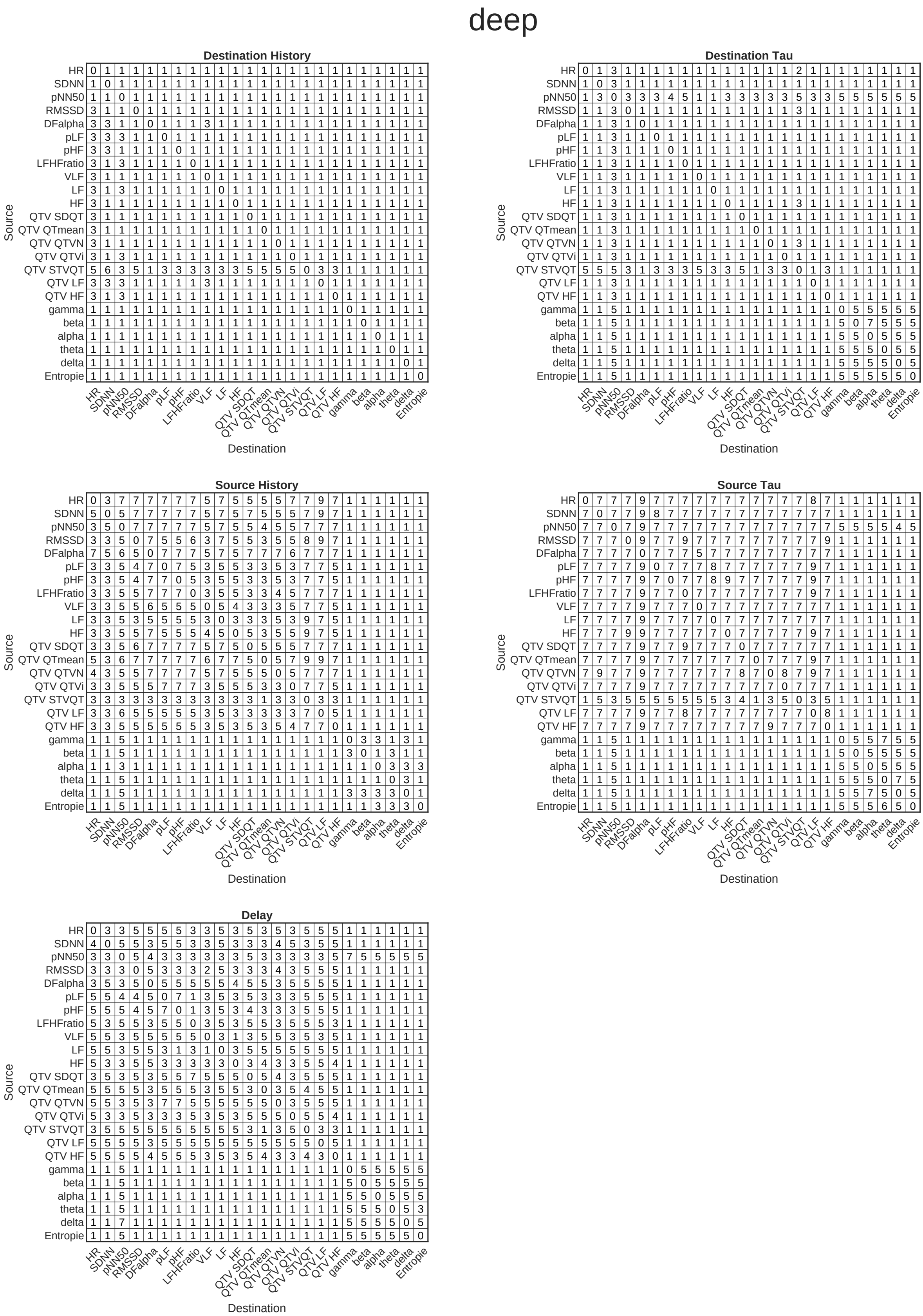

Figure S3. Embedding parameters during sleep stage: deep. Individual calculated for every source and destination parameter combination. Embedding parameters consists of history length and tau τ for source and destination separately and the delay between source and destination.

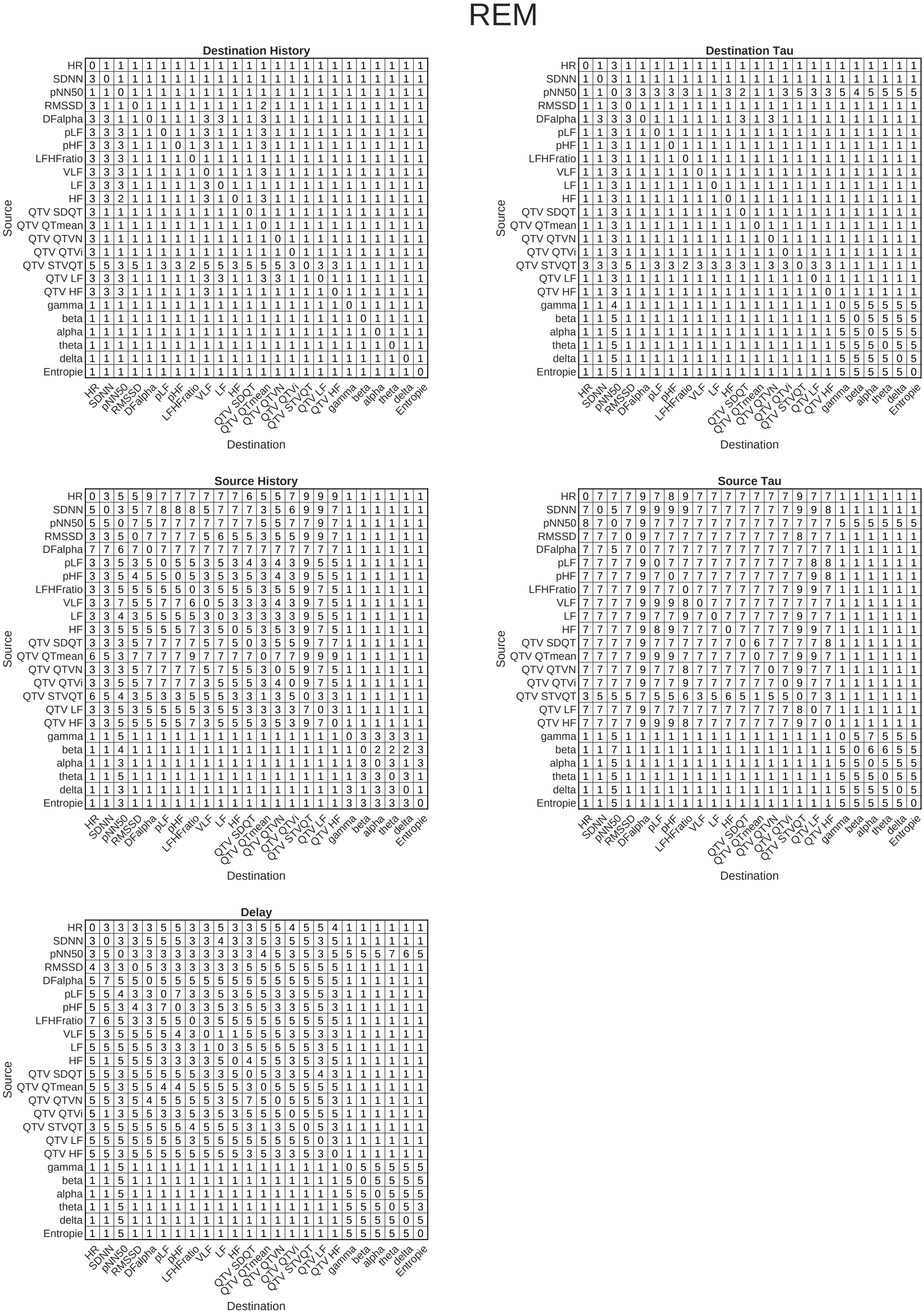

Figure S4. Embedding parameters during sleep stage: REM. Individual calculated for every source and destination parameter combination. Embedding parameters consists of history length and tau τ for source and destination separately and the delay between source and destination.
